# Fundamentals to clinical application of nanoparticles in cancer immunotherapy and radiotherapy

**DOI:** 10.3332/ecancer.2020.1095

**Published:** 2020-09-01

**Authors:** Vinodh Kumar Selvaraja, Deleep Kumar Gudipudi

**Affiliations:** Department of Radiation Oncology, Basavatarakam Indo American Cancer Hospital and Research Institute, Road number 10, Banjara Hills, Hyderabad 500034, Telangana, India; a https://orcid.org/0000-0002-2507-8328; b https://orcid.org/0000-0003-3447-4725

**Keywords:** nanoparticles, nanocarriers, cancer immunotherapy

## Abstract

Cancer immunotherapy has made rapid progress over the past decade leading to high enthusiasm and interest worldwide. Codelivery of immunomodulators with chemotherapeutic agents and radioisotopes has been shown to elicit a strong and sustained immune response in animal models. Despite showing promising results in metastatic and recurrent cancers, the utilisation of immunotherapy in clinical settings has been limited owing to uncertainties in elicited immune response and occurrence of immune-related adverse events. These uncertainties can be overcome with the help of nanoparticles possessing unique properties for the effective delivery of targeted agents to specific sites. Nanoparticles play a crucial role in the effective delivery of cancer antigens and adjuvants, modulation of tumour microenvironment, production of long-term immune response and development of cancer vaccines. Here, we provide a comprehensive summary of nanotechnology-based cancer immunotherapy and radiotherapy including basics of nanotechnology, properties of nanoparticles and various methods of employing nanoparticles in cancer treatment. Thus, nanotechnology is anticipated to overcome the limitations of existing cancer immunotherapy and to effectively combine various cancer treatment modalities.

## Introduction

Cancer immunotherapy, which utilises the body’s own immune system to fight against tumour cells, has grown in importance over the past decade. Novel strategies employed to enhance cytotoxic T-cell activation include chimeric antigen receptor T-cell therapy, immune checkpoint blockade (ICB) therapy and neoantigen vaccines [1–8]. Cancer immunotherapy has shown remarkable results in certain cancers which had poorer outcomes, especially in childhood acute lymphoblastic leukaemia, where CAR T-cell therapy targeting B-cell antigen (CD19) [9–13] led to an overall remission rate of 82.5% [14, 15], and in advanced melanoma, where cytotoxic T lymphocyte antigen 4 antibody improved the overall survival and 5-year recurrence-free survival [16, 17]. Compared to chemotherapy and radiotherapy, immunotherapy confers greater and long-term benefits in recurrence or metastatic setting [18–20]. Despite promising results, the effect of immunotherapy in solid tumours is less pronounced [21, 22] due to immunosuppressive tumour microenvironment and abnormal extracellular matrix created in cancer cells [23–25]. This is where biomaterial-based nanoparticles can play a crucial role in the effective delivery of immunotherapy.

## Fundamentals of nanoparticles

Nanoparticles are nanoscale materials, usually made up of polymeric, liposomal and metallic formulations [26]. An ideal nanoparticle should be low-toxic, biodegradable, highly specific and cost effective [27]. Nanoparticles deliver the intended product to target sites by three methods: passive targeting, active targeting and physical targeting. Passive targeting is based on enhanced permeability and retention (EPR) effects, in which tumour cells selectively absorb nanoparticles, whereas, in active targeting, nanoparticles are coupled with ligands which interact with receptors over expressed in target sites. Physical targeting utilises the optical, thermal and magnetic properties of nanoparticles, where external sources guide them to reach the specific target sites [28].

## Basis of nanotechnology application in cancer treatment

Nanotechnology-based drug delivery system has been utilised to enhance the efficacy of chemotherapy, radiotherapy and photodynamic therapy [29, 30]. The prime aim of using such systems is to enlarge the therapeutic window and the effective delivery of drugs [31]. Nanoformulations have shown improvement in a therapeutic window by acting at different levels of drug pharmacokinetics. The size of nanoformulations avoids renal clearance, and PEGylation prevents opsonisation by macrophages, thereby prolonging systemic circulation of drug [32]. The justification of cancer nanomedicine is based on the phenomenon called EPR effect [33]. The abnormal vasculature of tumour cells allows greater permeability, whereas the ineffective lymphatic clearance aids in the accumulation of nanoparticles inside tumour cells [34, 35]. Nanoparticles can also target cancer cells directly, either by coupling with a ligand and binding to the receptor of cancer cells (ligand–receptor complex) or by binding of surface moieties [36].

## Application of nanotechnology in cancer immunotherapy

Nanotechnology used in cancer immunotherapy targets not only cancer cells but also lymphocytes and antigen-presenting cells (APCs) in circulation, thereby helping to generate a robust immune response. Hence, a much lower concentration of drug is needed when used in conjunct with immunomodulators [37]. In virtue of their high surface area to volume ratio, they are capable of carrying high-density peptide-major histocompatibility complex (pMHC), which, in turn, fastens the re-engagement of dissociated pMHC, and thereby delays the internalisation of T-cell receptors and prolongs the time for antigen presentation [38]. Therefore, the enhancement of cross-presentation of neoantigen to antigen-presenting cells leads to greater immune response. Nanotechnology can be used to intervene at various stages of cancer immunity cycle [39]. It can be used in the delivery of neoantigens for cancer vaccine development, delivery of adjuncts to increase immunogenicity, modulate tumour microenvironment, enhancement of immune recognition, delivery of checkpoint inhibitors and codelivery of checkpoint inhibitors with costimulatory immunomodulators, in adoptive immunotherapy and image-guided immunotherapy [40].

### Cancer nano-vaccines

Cancer nanovaccines are designed for the effective delivery of tumour-derived protein antigens, or peptide antigens, or nucleic acid antigens to APCs, which, in turn, triggers an immune response [40]. Numerous research works are going on in the development of effective cancer nanovaccines. One group of researchers designed a sodium alginate-based formulations containing I-131-labelled catalases, which would transform into a hydrogel inside tumour cells in the presence of Ca^2+^(calcium ion), and demonstrated a loading cytosine-guanine oligodeoxyneucleotide (CpG ODN) with the radio-enhanced sodium alginate formulations resulted in strong systemic immune response [41]. Another group demonstrated the activation of dendritic cells, which depends on antigen association on nanoparticles and type of surfactants used. They reported absorption or encapsulation of antigens with nanoparticles, which increases expression of MHC Class II and MHC Class I, respectively, and observed a greater expression of CD86 by dendritic cells when nanoparticles are coated with polyvinyl alcohol (PVA) as surfactant rather than PF127. They concluded that the best antigen-specific T-cell response is produced with chitosan-mixed polylactide co-glycolide (PLGA) or polylactide co-glycolide block-polyethylene glycol (PLGA-b-PEG) formulation for ovalbumin antigens and CpG with PVA-coated, antigen-encapsulated nanoparticles [42].

Protein nanocarriers such as albumin–drug conjugates increase the half-life of drug in blood; potentiate draining to lymph nodes, enhance antigen presentation to APCs and thereby improve antitumour activity [40]. Abraxane is a nanodrug approved by the FDA, which is an albumin–paclitaxel conjugate used for advanced non-small cell lung cancer, metastatic pancreatic and breast cancer. Sahin et al. reported that the delivery of RNA-encoded antigens using DOTMA/DOPE liposomes led to a strong effector and memory T-cell responses along with IFN-alpha-mediated tumour rejection in animal models. A similar response was observed in their phase I trial too [43]. Nanovaccines can also be designed by coating natural cell membranes such as platelet membrane and leukocyte membrane onto a synthetic nanoparticulate core. The studies demonstrated that platelet membrane-coated nanoparticles selectively adhere to damaged vasculature and enhance binding to platelet-adhering pathogens, whereas leukocyte membrane-coated nanoparticles communicate with endothelial cells and transport payloads across inflamed reconstructed endothelium [44–46].

### Co-delivery of immune adjuvants

Nanoparticles also help in the co-delivery of immune adjuvants along with antigens to avoid immune tolerance [29]. The commonly used adjuvants in cancer immunotherapy are lipopolysaccharide, 3-O-desacyl-4′-monophosphoryl lipid A (MPLA), Toll receptor agonists such as CpG oligodeoxynucleotides (ODNs) and polyinosinic:polycytidylic acid (poly I:C), agonists of the stimulator of IFN genes (STING) and cytokines (e.g., IL-2 and GM-CSF) [47]. Adjuvants such as CpG ODNs, STING and poly I:C are negatively charged, whereas nanoparticles are positively charged, and hence, they form electrostatic complexes. By virtue of which, nanoparticles can aid in the effective delivery of these adjuvants into APCs along with tumour antigens, thereby promoting anticancer immune response [48, 49]. When the above mechanism is combined with ICB, there can be further enhanced anticancer immune response. A multifaceted immunomodulatory nanoliposomes developed by Lim group contain cancer membrane antigens to improve specificity towards tumour and two immunostimulatory adjuvants for immune stimulation such as MPLA and dimethyl dioctadecyl ammonium. This combination is denoted as tumosomes, which has shown to inhibit tumour growth and improve survival in mouse tumour models [50]. Nanoparticles can also deliver various anticancer therapeutic agents such as chemotherapy, immunotherapy and cell-based therapy to tumour sites, thereby improving therapeutic effect and minimising toxicity.

## Application of nanotechnology in cancer radiotherapy

Radiotherapy has been a vital component of cancer treatment for several decades. With the advent of modern radiotherapy treatment machines and planning systems, the therapeutic ratio of radiotherapy has significantly improved. However, there still exist many challenges that hinder the effective treatment of cancer with radiotherapy. The major limitations of radiotherapy are hypoxic tumours, less radio responsive/radioresistant tumours, increased toxicity to adjacent normal structures and side effects while combining with chemotherapy and immunotherapy. These shortcomings of radiotherapy can be overcome with the help of nanotechnology.

### Radioisotope delivery by nanocarriers

Radioisotopes used in radioimmunotherapy are broadly grouped into three categories based on the type of emitted particulate radiation. They are beta-emitters (^90^Y, ^131^I, ^199^Lu, ^186^Rh, ^89^Sr, ^32^P and ^67^Cu), alpha emitters (^213^Bi, ^221^At and ^225^Ac) and low-energy electron emitters (^125^I, ^67^Ga, ^111^Ir, ^99m^Tc and ^201^Th). Among the three, alpha particles have a high linear energy transfer and are more destructive. However, the therapeutic effect of radioisotopes is largely hindered by rapid renal clearance due to smaller size (5 nm) and opsonisation by mononuclear phagocyte system [51]. This problem is overcome by loading/conjugating radioisotopes to nanocarriers. Nanoparticles such as liposomes, micelles and polymeric complex decrease renal clearance owing to their increased size effect (10 nm) [52–55]. Furthermore, PEGylation of nanoparticles prevents the adsorption of opsonins due to steric hindrance produced by the presence of polyethylene glycol (PEG). Therefore, radioisotope-labelled nanoparticles significantly increase the short life of radioisotopes in blood. Wang *et al* [56] demonstrated that ^111^In and ^177^Lu PEGylated liposomes had a longer half-life in blood compared to ^111^In-DTPA in mice model. They also improve an intratumoural accumulation of radioisotopes through EPR effect and decrease the dose to surrounding normal structures, thereby improving therapeutic ratio [57, 58]. A PEGylated liposomal formulation of doxorubicin has a much slower clearance rate and an AUC 300 times greater than with free doxorubicin. It also proved to have greater intratumoural drug concentration and prolonged exposure time compared to free doxorubicin [59].

### Nanoformulations of radiosensitizers

Hypoxia is a major deterrent of radiation effects on tumour cells [60, 61]. Various strategies have been tried to overcome hypoxia and improve tumour cell kill to radiation, one among them is the usage of radiosensitizers [62]. Radiosensitizers are agents, which when used in conjunction with radiotherapy improve its lethal effects on tumour. An ideal hypoxic cell radiosensitizer must be chemically stable, highly soluble in water or lipids and more importantly selectively target tumour hypoxic cells sparing normal tissues. Various drugs of nitroimidazole group such as metronidazole, misonidazole, etanidazole and nimorazole have been tried as radiosensitizers [63]. Their efficacy as radiosensitizer is greatly reduced by dose-limiting toxicities and less solubility [65–67]. However, nanoformulations of radiosensitizers have shown to improve the effective delivery of these agents to tumour sites. In a study on mice model, nanoformulation of wortmannin (phosphatidylinositol 3ʹ kinases & related kinases inhibitor), composed of DSPE-PEG (1,2 distearoyl-sn-glycero-3-phosphoethanolamine - polyethylene glycol) lipid shell and PLGA polymer core, has shown to be more effective than 5FU as radiosensitizer [68]. Similarly, nanoformulations of histone deacetylase inhibitor has proved to be an effective radiosensitizer used in colorectal and prostate cancer cell lines [69, 70]. Nanoformulation-based radiosensitizers have a sustained DNA repair inhibition effect and achieve a lower concentration in normal tissues [70, 71].

In addition to acting as a carrier, few nanomaterials of high atomic number such as gold (*Z* = 79) and gadolinium (*Z* = 64) can act as potential radiosensitizers as dose absorbed by tissue is proportional to *Z*^2^ of the material. The study by Zhang *et al* [72] demonstrated that tumour inhibition by radiotherapy can be significantly improved by using ultrasmall glutathione-coated gold nanoclusters as radiosensitizers. Combining radiation with gadolinium-based nanoparticles as radiosensitizers led to a significant tumour growth delay in a mouse model [73].

### Usage of nanoparticles to overcome radioresistance

Radioresistance is another major factor leading to treatment failure after radiotherapy [74]. Apart from inherent less radiosensitivity of few tumours, it is the presence of hypoxia with central necrosis, expression of DNA repair enzymes and anti-apoptotic proteins that lead to radioresistance [75, 76]. Nanotechnology helps in reducing radioresistance by targeting the related signalling pathways and genes. In the preclinical study on tumour-bearing mice model, the administration of bevacizumab 48 hours before radiotherapy led to the normalisation of tumour vasculature leading to temporary tumour reoxygenation and better radiosensitivity [77]. Another approach using nanoformulations of small interfering RNA (siRNA) and radiotherapy has shown to have encouraging results in overcoming radioresistance. On combining iron oxide nanoparticles coated with PEG and PEI to siRNA, LD50 of irradiation was reduced by threefold in medulloblastoma and ependymoma cells [78]. A similar combination of siRNA and PEG-PEI copolymer against sCLU protein drastically reduced cell survival after 0.5 and 3 Gy compared to radiotherapy alone group in breast cancer cells *in vitro* [79]. Zheng *et al* [80] recognised TRAF2 (TNF receptor-associated factor 2) as a potential target for siRNA silencing in glioblastoma cells, which led to radiosensitisation and tumour growth suppression.

### Nanotechnology to combine RT and other treatment modalities

In the majority of locally advanced cancers, a multimodality treatment approach is advocated, especially chemoradiation [81]. Chemotherapy acts as a synergistic to radiotherapy and radiosensitizer [82]. However, the combination therapy leads to increased toxicity. Nanotechnology can help to alleviate this problem by the selective delivery of chemotherapeutic agents to tumour sites, thereby reducing toxicity during combination therapy. In addition to their synergistic effect, certain chemotherapeutic agents such as cisplatin, paclitaxel and doxorubicin also act as radiosensitizers [83–85]. In a tumour-bearing mice model, cisplatin delivered with upconversion nanoparticle led to enhanced effects by the release of both cisplatin and high metal ions [86]. The studies showed that the combination of chemotherapy and radiosensitizers in a single nanoparticle yields better chemotherapeutic effect than when loaded in separate nanoparticles. Au *et al* [87, 88] combined docetaxel and wortmannin in PLGA nanoparticle in an *in vivo* study and demonstrated that the toxicity profile of nanoparticle combined formulations had lesser hepatotoxicity and hematologic toxicity in comparison to the administration of each drug alone.

Another interesting prospect is targeted nanoparticle, in which the efficiency of nanoparticles is increased by surface modification of targeted ligands. Commonly targeted ligands are RGD peptide, folate and transferrin [89–91]. An *in vivo* study with docetaxel-loaded, folate-conjugated nanoparticle showed that targeted nanoparticle is more efficient as radiosensitizer compared to nanoparticle without targeting ligands [92]. In a similar study, folate-targeted nanoparticle loaded with paclitaxel and yttrium-90 as a combination therapy proved to be superior in a murine model with ovarian cancer with peritoneal metastasis [93].

## Nanotechnology-based image-guided cancer immunotherapy and radiotherapy

Image-guided cancer immunotherapy is another aspect of immunotherapy, where inorganic/metallic nanoparticles are used not only to deliver tumour antigens but also to provide imaging contrast for theranostics and immunogenic cell death through heat-induced or reactive oxygen species [94]. Researchers developed an iron oxide–zinc oxide core–shell nanoparticle, where the zinc oxide surface binds to certain peptide motifs with high binding affinity, whereas the iron oxide core provides imaging contrast for monitoring the migration of the nanovaccine as well as the activated DCs with magnetic resonance imaging (MRI) [95].

Gold nanoparticles have a multiple utility in cancer treatment such as signal enhancer for CT-guided radiotherapy, radiosensitizer and agents for photodynamic therapy [96, 97]. They are also used as CT contrast agents to assess the response to immunomodulators [98]. Poly N-isopropyl acrylamide-coated gold nanoparticles in a gel matrix of sucrose acetate isobutyrate/EtOH/PLA (ethyl alcohol/polylactic acid) developed by Anderson *et al* [99] proved to be an excellent liquid fiducial tissue marker providing high-resolution micro-CT images in mice model for 2D X-ray visualisation. The same when used in canine cancer patient provided an enhanced image contrast for 2D X-ray and CT imaging with no side effects. In a mice model with intracerebral malignant gliomas, image-guided radiotherapy was delivered along with gold nanoparticles, where micro-CT images showed 19-fold higher intratumoural uptake of gold nanoparticles compared to normal brain [100].

Gold nanoparticles are also used as a part of nanocomposite, which contain two nanoparticles. Multimodal imaging with such nanocomposite can provide more information for accurate radiotherapy treatment delivery. In a tumour-bearing mice model, PCL-PEG micelle system loaded with SPIO and gold nanoparticles showed a selective accumulation in tumour and enhancement of tumour margins in MRI. Furthermore, the above nanocomposite improved 90-day survival rate [101]. Gold nanoparticles are also helpful in radiation *in vivo* dosimetry. Researchers developed a nanosensor composed of liquid surfactant-templated formation of coloured dispersions of gold nanoparticle, which can detect radiation dose from 0.5 to 2Gy. It provided the qualitative and quantitative assessment of radiation through the naked eye and absorbance spectrophotometer, respectively [102]. Nanosensors with upconversion nanoparticles and oxygen indicator have high sensitivity and specificity for the detection of oxygen changes in hypoxic environment and are useful in hypoxia imaging [103].

## Challenges and future directions

After having successfully studied the properties and feasibilities of utilising nanoparticles in cancer immunotherapy through animal models, the biggest challenge lying ahead is translating these preclinical technologies into clinical practice. Clinical trials on combining immunotherapy and radiotherapy are already being conducted worldwide. Combining stereotactic body radiotherapy and nanoparticle-mediated dendritic cell activation resulting in ablation of tumour cells and nanoparticle-guided photodynamic therapy providing a high precision treatment are potential areas to be explored in the treatment of solid tumours. The other major roadblock is that we are yet to fully understand the toxicity profiles of nanoparticle-mediated immune response. It is unclear whether the codelivery of immunotherapeutic agents with chemoradiotherapy will result in intolerable side effects. Therefore, it is mandatory to study the toxicity profiles of nanoparticles and its modulation with various components of nanoformulations. With an improvement in understanding of cancer biology and cancer research, nanotechnology-based immunotherapy will change the treatment methodology and prognosis of advanced malignancies such as glioblastoma multiforme and pancreatic cancer in the near future.

## Conclusion

With increasing global research on cancer immunology and biomedical engineering, the uncertainties surrounding combining nanoformulations of cancer immunotherapy and radiotherapy will be answered in the coming decade. Nanotechnology-based cancer immunotherapy and radiotherapy will soon turn out be a golden sword in the armory of every oncologist in the fight against cancer.

## Conflicts of interest

There is no conflict of interest to declare.

## Author’s contribution

The first author (VKS) contributed in the conception of this review article, literature review and manuscript writing. The second author (DKG) contributed with his suggestions, proofreading and supervision.

## Funding statement

No organisational grants or support was obtained for writing this manuscript.
